# Towards a unified framework to study causality in Earth–life systems

**DOI:** 10.1111/mec.16142

**Published:** 2021-09-06

**Authors:** Greer A. Dolby

**Affiliations:** ^1^ School of Life Sciences Arizona State University Tempe Arizona USA; ^2^ Center for Mechanisms of Evolution Arizona State University Tempe Arizona USA

**Keywords:** causal theory, diversification, Earth–life science, evolution, macroecology, theory

## Abstract

There is considerable interest in better understanding how earth processes shape the generation and distribution of life on Earth. This question, at its heart, is one of causation. In this article I propose that at a regional level, earth processes can be thought of as behaving somewhat deterministically and may have an organized effect on the diversification and distribution of species. However, the study of how landscape features shape biology is challenged by pseudocongruent or collinear variables. I demonstrate that causal structures can be used to depict the cause–effect relationships between earth processes and biological patterns using recent examples from the literature about speciation and species richness in montane settings. This application shows that causal diagrams can be used to better decipher the details of causal relationships by motivating new hypotheses. Additionally, the abstraction of this knowledge into structural equation metamodels can be used to formulate theory about relationships within Earth–life systems more broadly. Causal structures are a natural point of collaboration between biologists and Earth scientists, and their use can mitigate against the risk of misassigning causality within studies. My goal is that by applying causal theory through application of causal structures, we can build a systems‐level understanding of what landscape features or earth processes most shape the distribution and diversification of species, what types of organisms are most affected, and why.

## 1 INTRODUCTION

Imagine a version of Earth in which the movement of tectonic plates builds mountains and topography, but where there are no geological hotspots forming island archipelagos, no methane levels fluctuating through time, and no growing and shrinking of glaciers. In a world where there is only growth of topography, what does the diversity and distribution of life look like? This may be an unfair question as any geologist would point out that it is not possible to separate geological processes in this way; as soon as topography grows, rivers flow through and **incise** that topography to form ridges and valleys. In fact, steeper slopes drive higher rates of river **incision**—a relationship governed by the stream power equation (Whipple & Tucker, 1999). Yet, I would argue that this is the basis for much of what phylogeography aims to achieve—to understand how individual geological and climatic (geoclimatic) processes shape the distribution and the diversification of life on Earth. The same is true of studies that use phylogenetic or species richness (macroecological) data coupled to features of the landscape, such as mountains or latitudinal gradients (Antonelli, Kissling, et al., 2018; Hoorn et al., 2010; Rabosky et al., 2018; Rahbek, Borregaard, Antonelli, et al., 2019).

Within statistical and comparative phylogeography, a major goal is to understand what geoclimatic factors govern the evolution and distribution of populations, whether species respond similarly or not, and why (Crandall et al., 2019; Dolby et al., 2015, 2019; Leaché et al., 2020; Myers et al., 2016; Thomaz & Knowles, 2020; Wan et al., 2021). Here, I explain how answering these questions is really about establishing causal relationships between the nonliving yet changeable landscape and species which evolve in response to it. Of course, there are many biological/intrinsic factors that also drive species diversification, such as differential adaptation (Chapman et al., 2013; Favre et al., 2017; Tobler et al., 2018), disruptive sexual selection (Hudson & Price, 2014; Martin & Mendelson, 2016; Servedio & Boughman, 2017), polyploidization (Wood et al., 2009) and niche specialization (Deng et al., 2019; Ford et al., 2016; Gharnit et al., 2020). However, in this article, I will focus specifically on the physical landscape and try to show that causal theory fits naturally into answering phylogeographical and macroecological questions. To do this I will first present evidence for why earth processes can be thought to impart an organized, deterministic effect on species evolution. Then, I will show that the landscape features earth processes produce, and which are commonly studied, are aggregations of variables whose effects can be teased apart using a set of tools called causal structures (*sensu* Grace et al., 2012). These tools represent causal hypotheses as networks and can be used to organize and restructure knowledge from individual studies to build Earth–life theory and guide new hypotheses, as I will demonstrate with examples from the literature.

## 2 IS SPECIATION DETERMINISTIC?

**FIGURE 1:**
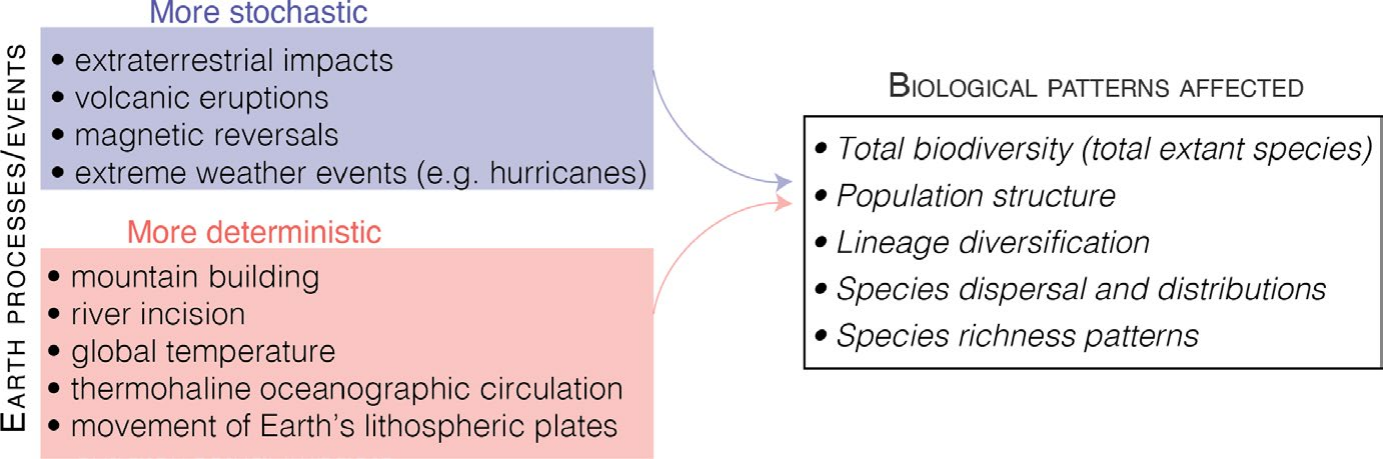
Diagram showing examples of mostly stochastic and mostly deterministic earth processes or events that can impact biology. Determinism here refers to processes whose outcomes can be moderately well predicted from initial starting conditions and knowledge of the system (i.e., quasideterministic). Stochastic events are those which are poorly predicted in time and space or which have a random distribution of effects on biology. Note that stochastic vs. determinism here is considered a gradient, where these processes do not fall perfectly into one group or the other. For example, global temperature can be estimated from greenhouse gas concentrations, but a component of those concentrations are stochastically driven (i.e., from volcanism)

Scientists have long debated how predictable life is (Kolata, 1975). In 1989, Gould introduced a now‐famous thought experiment about replaying the tape of life, that is, if life were restarted from the beginning, would it result in the same outcome we see today (Gould, 1989, pp. 48–49)? Gould and others argued that life is unrepeatable because it is the product of initial starting conditions and random stochastic events (Blount et al., 2018; Gould, 1994; Raup et al., 1973; Schopf et al., 1975). For example, if life started over, there might be different mass extinction events. Or, mutations that were key to evolutionary transitions in our history may arise at a different time point and so affect a different set of organisms, or they might not arise at all. Others suggested that life is the “inevitable” product of channellizing forces such as the favourability of certain chemical reactions, or of developmental constraints engrained early on that make some biological outcomes likely to be repeated again and again (Flessa & Levinton, 1975; Morris, 2006, 2010). Debated often at a macroevolutionary level, the theme echoes at the molecular level with studies of parallel evolution (Powell & Mariscal, 2015). Work on sticklebacks has shown that genetic mutations in the same genes are responsible for the repeated loss of armoured plates as fish colonize low‐predator streams (Colosimo, 2005; Schluter & Clifford, 2004). The *mc1r* gene has been shown to underpin divergence in coat and plumage colour in many independent species and ecological settings (Brockerville et al., 2013; Mundy, 2005; Ritland et al., 2001; Steiner et al., 2007). Finally, the specialization of *Anolis* lizards into ecological microniches has been repeated across the *Anolis* phylogeny (Gunderson et al., 2018; Losos et al., 2003; Velasco et al., 2016). Repeated molecular or phenotypic evolution is not equivalent to repeating all of life's diversity over the last billion years. To this point, however, the stochastic vs. deterministic debate has largely omitted one observation: species evolve in response to the physical landscape, and the earth processes that shape that landscape are themselves largely deterministic (Figure 1). So although the impact of a meteor may prune the evolutionary tree at random, the everyday processes that shape the landscape life lives on have behaved in a consistent way for much (if not all) of life's history. More explicitly, they may have an organized or deterministic influence on life even if life is not determinable itself (Smith & Morowitz, 2016).

There are many examples of deterministic behaviour among earth processes. When continental plates converge, they form mountainous topography and the height of this topography can be estimated from the shear‐force of the colliding plates (Dielforder et al., 2020), although climate‐modulated erosional processes may also be a major control (Brozovi et al., 1997; Champagnac et al., 2012; Egholm et al., 2009). The rate of river incision can be estimated from the stream power equation and rates increase with the amount of discharge and steepness of the surrounding slopes (Whipple & Tucker, 1999). The increase in mean global temperature and loss of ice volume on Earth can be predicted from the amount of methane and other greenhouse gasses that are added to the atmosphere (e.g., TFE.3 in Stocker et al., 2013). Some details of exactly how these processes unfold are still debated among Earth scientists. However, these examples show that the outcomes of geological and climatic events can be estimated to a *first order* and the large‐scale outcomes behave quasideterministically on the biological scales discussed here.

On the biological side, it is well established by theory and empirical studies how reduction of gene flow or adaptation to different selection regimes can cause lineages to diverge (Coyne & Orr, 2004). Adapting Gould's experiment, if we imagine that a mountain range is built 100 times over within the range of a low‐dispersing beetle, then with all else equal we might expect that if that beetle lineage diverges due to isolation in one iteration then it would diverge in isolation in many other iterations. In contrast, we would expect that distribution of outcomes to differ if we performed the same 100 experiments using a high‐dispersing bird species. This is because we know these organisms have vastly different traits. These two outcomes are probabilistic, not deterministic, because the outcome of any trial is not perfectly predictable. However, if we agree that earth processes behave quasideterministically and the divergence response of organisms is likely to vary based on a set of biological traits, then it stands to reason that we should be able to build a set of “speciation boundary conditions” that describe what geological settings promote the origination of lineages and amongst which groups. The main challenge becomes measuring individual cause–effect relationships between earth processes and evolutionary patterns. Although this is what many phylogeographical studies seek to do, we lack an organizing framework to systematically compare individual taxonomic and geographical studies to achieve this greater synthesis. I believe one path forward is through using causal structures, particularly in more deterministic scenarios (Figure 1), which I will introduce and apply in the next sections.

## 3 CAUSALITY IN OTHER FIELDS

**FIGURE 2:**
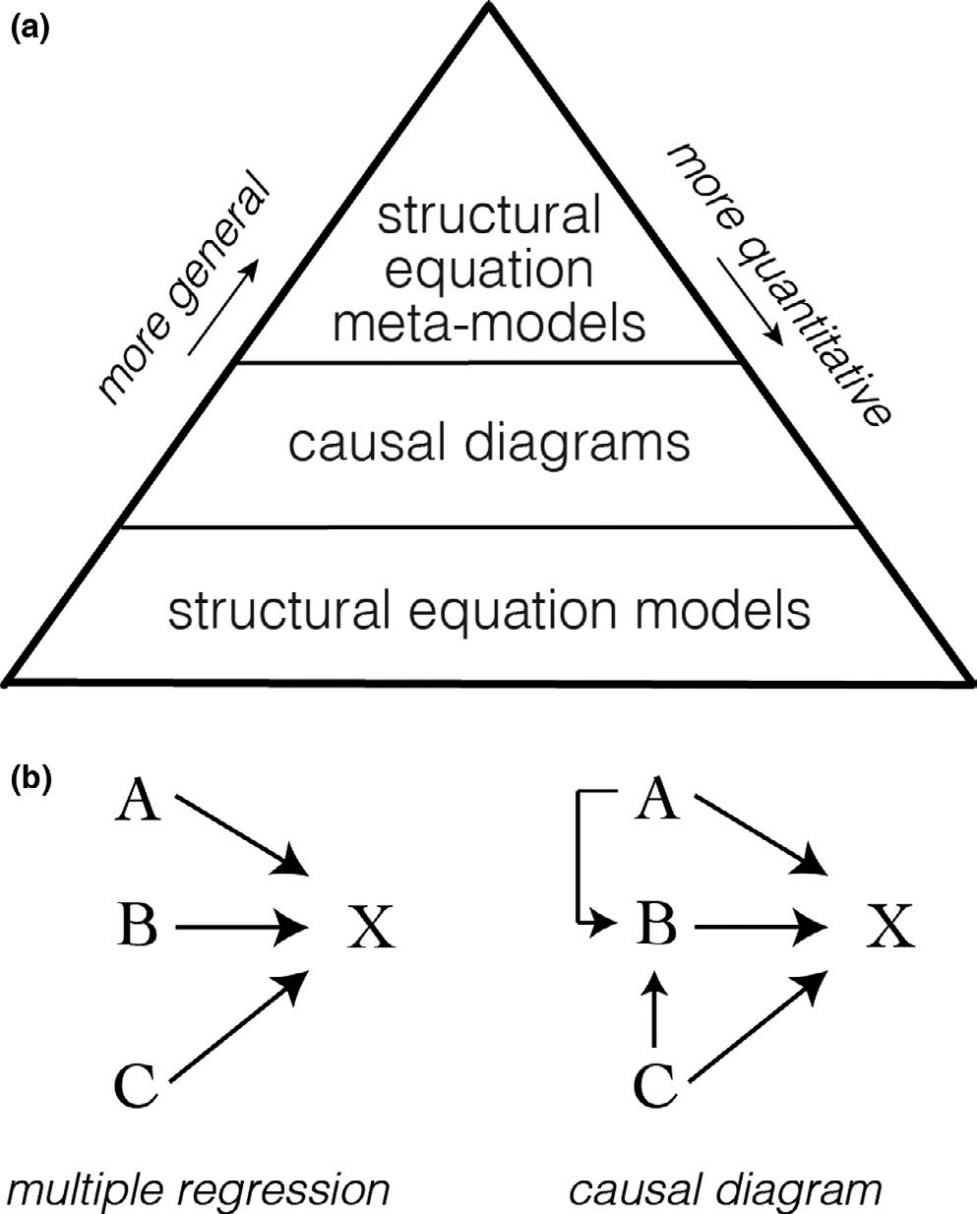
Summary of causal structures. (a) Causal structures defined in Grace et al. (2012) from most general (top) to most specific (bottom). Structural equation meta‐models (SEMMs) are conceptual networks that describe the higher level, generalizable theory of a system. Causal diagrams (CDs) are more specified and serve to bridge higher level theory to a study of interest; CDs play a pivotal role in translating theory to the design and interpretation of a study and vice versa. The most detailed level are structural equation models (SEMs) that convey the variables measured in a study, their causal relationships and the statistics used to quantify its paths. (b) An example of how a multiple regression (A, B, C onto X) could be translated into a causal diagram that would support compound pathways (A → B → X) to allow for more nuanced depiction of a system. In this example, by not allowing compound pathways the multiple regression might overemphasize the importance of variable B on X relative to the causal diagram, or the role of B may be oversimplified

Judea Pearl largely formalized the algorithmic and mathematical definition of causality (Hopkins & Pearl, 2007; Pearl, 1995, 1998, 2009; Pearl & Verma, 1995), which is key for modelling systems in a way where new knowledge can be learned beyond what is already observed. Pearl and Verma wrote, “… an intelligent system attempting to build a workable model of its environment cannot rely exclusively on preprogrammed causal knowledge, but must be able to translate direct observations to cause‐and‐effect relationships” (Pearl & Verma, 1995). Causal theory has since been applied widely across disciplines, for example within the social sciences especially when variables are intangible or difficult to measure (e.g., intelligence; see references in Pearl, 1995), as well as within artificial intelligence (Pearl, 2019). Grace and colleagues have done tremendous work to adapt causal theory for use in ecological studies (Eisenhauer et al., 2015; Grace, 2006, 2010, 2015; Grace et al., 2010; Grace & Bollen, 2007; Grace & Irvine, 2020; Pugesek & Grace, 1998). A key development of this work was the translation of causal principles to be used in observational studies, whereas Pearl's original theory was developed specifically for interventionist experiments (i.e., laboratory experiments) where variables can be controlled and manipulated to establish and quantify “true” causal relationships (Pearl & Verma, 1995). However, through knowledge of a study system and careful development of causal structures (graphs) at different levels of detail, these ecological studies have relaxed this interventionist constraint; results from observational studies are then not interpreted strictly as causal inferences, but instead as *estimates* of causal relationships. Despite this more limited interpretability, the use of causal structures in ecological studies has contributed substantive new knowledge about system dynamics in several settings (Eisenhauer et al., 2015; Grace, 2010; Grace et al., 2016). Within evolutionary biology, use of causal structures has been limited to the application of structural equation models to quantify genotype–phenotype relationships (Li et al., 2006; Otsuka, 2014; Scheiner et al., 2000). Here we will use the two higher order causal structures—structural equation metamodels and casual diagrams (Figure 2; Grace et al., 2012)—to bridge the earth sciences with evolutionary biology.

## 4 IMAGINING CAUSALITY FOR EARTH–LIFE SCIENCE

**FIGURE 3:**
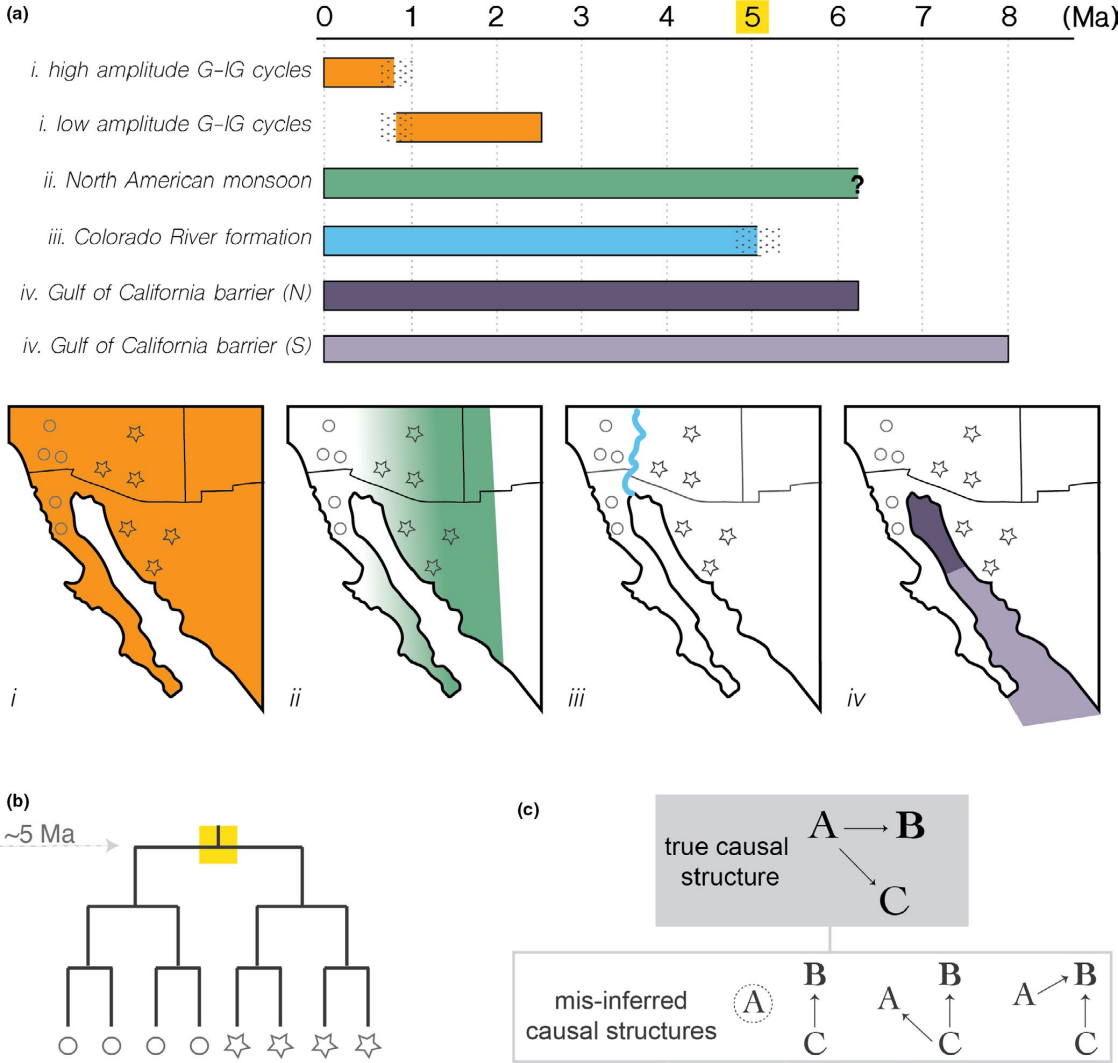
An example of how co‐occurring geoclimatic processes can interfere with the ability to accurately identify which changes in the landscape initiated a biological pattern of interest, in this case speciation of lineages in the southwestern USA. (a) Depiction of the geoclimatic events thought to have occurred over the time period when most lineages diverged (circle vs. star; Dolby et al., 2019). Stippling represents boundary uncertainty, and a question mark denotes a boundary of unknown age. Panels i–iv show cartoon representations of the geographical extent of each process. (b) A toy phylogenetic tree to show the pattern of lineage divergence for desert tortoises (Edwards et al., 2016); note that a divergence time of 5 million years roughly correlates with three of the four major processes (monsoon, river formation and flooding of the Gulf of California in (a)). (c) Causal diagrams showing how a true causal relationship can be misinferred due to collinear variables if not all variables relevant to the system are considered. Arrows depict causal relationships. The dotted circle represents an unsampled variable

As discussed in the beginning of this article, Earth's landscape is dynamic and shaped by many processes. This presents two challenges when working to link evolutionary patterns with underlying geological process(es). The first is that earth processes are interrelated and are therefore often co‐occurring (Figure 3a). Looking into the landscape history at many (perhaps most) locations on Earth will reveal that several aspects have changed over a given evolutionary period. The co‐occurrence of processes means that using the age of an evolutionary event (e.g., a lineage divergence or bottleneck) is *insufficient* to discern which aspect of the changing landscape caused a pattern (Dolby et al., 2015, 2019), a phenomenon known as **pseudocongruence** (Feldman & Spicer, 2006; Lapointe & Rissler, 2005; Riddle & Hafner, 2006; Soltis et al., 2006). In such cases, population genomic data have a benefit over phylogenetic data because they provide information not only in a spatial dimension that matches the spatial nuance of the landscape, but can be assayed for population effects and signs of local adaptation, particularly when whole genome data are used. Because some types of landscape change (e.g., differences in precipitation due to **monsoon** or **precession cycles**) are expected to drive adaptive divergence, and physical barriers may be expected to produce more “neutral” or nonadaptive divergence, the ability to interrogate both neutral and functional elements of the genome is potentially powerful. In this approach it is the structuring of types of information spatially between genetic and landscape features, rather than the coincidence of similar timings, that causally link the two systems.

The second (but related) challenge to linking geological processes with evolutionary patterns is that many of the most noticeable physiographic features of the landscape are in fact aggregations of **collinear** variables. Examples of aggregate features include mountain ranges, latitude and **bathymetry** that in the literature are long thought to control diversification and species richness patterns (Colwell & Hurtt, 1994; Hodkinson, 2005; Hoorn et al., 2010, 2013; McClain & Etter, 2005; Rabosky et al., 2018; Stevens, 1989). It is almost certain that these features *are* causal to the generation and/or distribution of biodiversity. However, it is the collinearity of direct variables such as temperature, precipitation and **solar insolation** within these aggregate features that makes it difficult to determine which of the variables exert causal control over a biological pattern (Rahbek, Borregaard, Antonelli, et al., 2019; Table 1).

**TABLE 1:** Linking aggregate features (left) with their direct causal mechanisms (right)

Aggregate features/phenomena	Constituent (direct) variables
Latitude	Temperature
	Insolation
Seasonality	Daylight
	Temperature
Mountain	Precipitation
	Soil type
	Temperature
	*p*O_2_
	Insolation
	Physical isolation (ruggedness)
Hydrothermal vent	Nutrient availability
	Temperature
	pH
Ocean bottom water	Nutrient availability
	Temperature

Note

These direct manifest (observable) variables are often easier to measure on the landscape and therefore their causal relations can be tested in different taxonomic and geographical settings.

**FIGURE 4:**
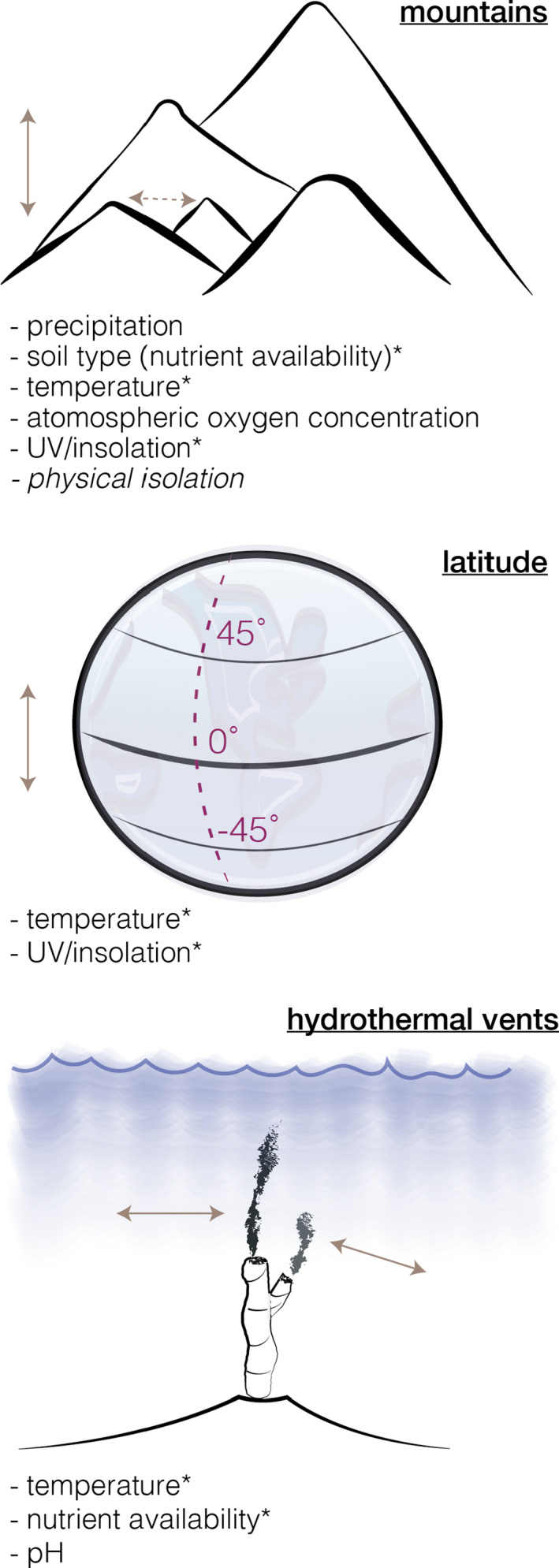
Cartoon examples of aggregate features on Earth. The direct variables associated with each feature are listed below and the arrows represent general axes over which the variables are expected to vary. Physical isolation (italicized) is expected to operate on a different axis than others (dotted line). Direct variables that exist in more than one feature have an asterisk. Using “natural experiments” (*sensu* Dawson, 2014) allows researchers to study the effect of different instantiations (i.e., occurrences) of direct variables

To give an example, if formation of a mountain range leads a lineage to diverge, is that divergence due to differential adaptation to the gradient in atmospheric oxygen or UV burden or temperature? Or was it because the lineage was physically isolated by peaks or valleys? The distinction here matters because if it is due to a temperature gradient then there are many other instantiations of that gradient on Earth, such as across latitudes or from hydrothermal vents (Figure 4). It may seem trivial but pinpointing the direct variable in this case would inform not only what we understand the external agents shaping evolution in the setting to be but also would direct what hypotheses to test in other settings to determine that variable's impact more broadly. If we return to the example of lineage divergence over a mountain, there is yet a trickier issue to contend with. In this example, if it was instead found that a lineage diverged due to physical isolation by ridges or valleys, would we say the mountain is causal to that divergence? Or are the rivers that did the work incising topography to form those ridges and valleys causal? Or is climate causal because, in a region devoid of rainfall, there would be no water to flow into streams to incise the topography? Or are they causally inseparable? There may not be a simple answer, but in the next sections we will see how causal structures can be used to represent complex networks of interactions to aid our thinking and discussion of complex causal networks.

### 4.1 The problem of epiphenomena

An **epiphenomenon** is a byproduct or an associated effect of the phenomenon of interest. Examples of epiphenomena mentioned above include temperature, precipitation and solar insolation, which are direct variables found in different collinear combinations within aggregate features (Figure 4; Table 1). The relevance of epiphenomena when working to establish causal relationships is clear: epiphenomenal variables can be easily confused with true causal variables and lead to spurious inferences (Gould & Johnston, 1972). If *A* is causal to *B* and *C* co‐occurs or covaries with *A*, then it may be incorrectly inferred that *C* is causal to *B*; or, that *C* is causal to *A* and *B*, or both *A* and *C* are causal to *B* (Figure 3c). This is particularly problematic when variable *C* is easier to observe or measure on the landscape than variable *A* in which case *A* may be overlooked.

In many statistical tests, including those common to phylogeography or macroecology (e.g., testing isolation by distance or spatial associations), often “no pattern” (randomness) is used as a null hypothesis. However, we know that the distributions of species or relatedness of populations is rarely, if ever, truly random. This poses a particular risk in the context of epiphenomena and pseudocongruence. If more than one aspect of the landscape has changed in a study region, or there is collinearity amongst direct variables, then if not all relevant features or variables are tested it is possible that whatever pattern detected, being nonrandom, will be interpreted as support for the experimental hypothesis even if it is not the “true” causal variable. This concept is well established, as many researchers have emphasized the importance of thoughtful hypothesis testing (Hickerson, 2014; Peterman & Pope, 2021). However, it becomes even more critical in the context of complex geoclimatic settings and when working to establish causal relationships between earth processes and evolution. For instance, vicariant barriers are often more obvious features on the landscape than ecological or climatic factors. In the western USA and Mexico, it was thought for decades that river and seaway barriers drove diversification of dozens of desert species, but recent work has highlighted the importance of less visible climatic phenomena as perhaps equally or more impactful (Dolby et al., 2015, 2019; Ornelas et al., 2018; Valdivia‐Carrillo et al., 2017). The reason these findings are important is *they change the hypothesized causal structure*—they shift our understanding of what processes are important for shaping species diversification or distributions (Figure 3). One comes to quickly appreciate how misassigning causality in many smaller individual studies can bias our understanding of the external controls on species diversification and distributions more broadly. When contending with epiphenomena, considering complexity of the geoclimatic setting is paramount; causal structures can help diagram these complexities.

### 4.2 An introduction to causal structures

**FIGURE 5:**
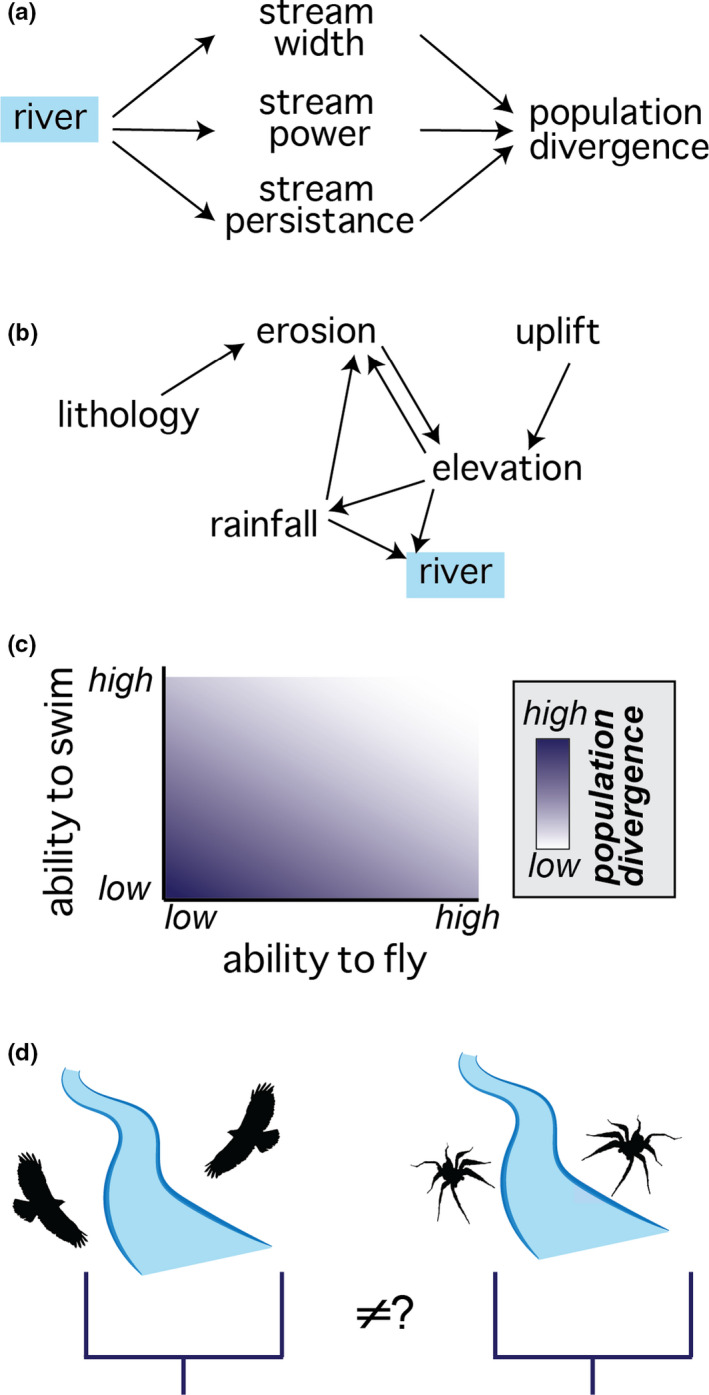
Depiction of how a river's impact on population divergence would depend on characteristics of the river as well as traits of the organisms. (a) Causal diagram of a river's general, measurable characteristics that might affect gene flow. (b) A more detailed causal diagram showing the suite of environmental, geological and climatic factors that would affect the river's traits depicted in the diagram above. (c) A graph showing the expectation that a river would be a stronger barrier to some types of species (e.g., water‐fearing or those which do not swim) than others, for example (d) it would be expected that a high‐dispersing bird and aqua‐phobic spider would have different levels of divergence associated with the same river, which should be determinable based on the characteristics shown in (a)

Before defining causal structures in detail, let us explain how they help to meet the challenges outlined in the last section. There is an increasing need for new theory to bridge the earth and life sciences (Antonelli, Ariza, et al., 2018; Rahbek, Borregaard, Colwell, et al., 2019). A primary strength of causal structures is that they simultaneously facilitate data analysis and theory development by forcing an explicit consideration of the variables relevant to a system and, importantly, their *relationships* (Grace et al., 2012; Pugesek & Grace, 1998). Causal structures are represented via **directed acyclic graphs** (Pearl, 1995, 1998) and depict cause–effect relationships at different levels of detail (*sensu* Grace et al., 2012) that serve different purposes. Causal structures rely on the visual representation of concepts or variables as networks (Figure 2b), which allows for the direct comparison of these relationships across different systems or studies. For instance, there are countless studies that test whether a river acts as a barrier to gene flow (Balao et al., 2017; Dolby et al., 2019; Lugon‐Moulin et al., 1999; Naka & Brumfield, 2018; Peres et al., 1996; Vechio et al., 2020; Weir et al., 2015) and these studies are necessarily carried out in different geographical areas, in different habitats and on different organisms, and the data then analysed in different ways. Meta‐analyses can be useful but are better for understanding whether there is statistical support for a general pattern, and in doing so, sacrifice the subtleties of individual studies. Yet, these subtleties are important. Causal structures instead embrace the nuance of individual studies in a way that can be systematically compared. In fact, it achieves a slightly different goal. A meta‐analysis might answer the question, “Do rivers structure populations?” Whereas, by comparing causal structures across studies one is instead asking, “*Under what conditions* do rivers structure populations?” Intuitively, the answer will depend on the characteristics of the organism, the characteristics of the river and perhaps on other factors (Figure 5). The subtle reframing of this question is not trivial—it opens the door to developing a richer and more mechanistic understanding of the role rivers play in evolution—which is not “yes” or “no” but rather some mathematical function or set of rules that describe “yes, under *these* conditions” (Figure 5). In addition, causal structures help make variables explicit, and therefore aid a researcher's task in formalizing and identifying potentially pseudocongruent variables. Even if such variables are not tested in the study, their identification can help others interpret the study's findings or guide the design of new studies. Presenting hypotheses as causal structures in publications would make it easier to identify what variables or relationships were tested in a study, identify which were excluded, and compare results of one study to another.

Importantly, causal structures also facilitate cross‐discipline collaborations and serve as a teaching tool for students. Formalizing these structures fosters productive discussion because these networks are a natural point of collaboration for geologists and biologists to discuss whether their knowledge is adequately represented in the model. The community is then strengthening cross‐disciplinary collaboration while engaging in theory development about Earth–life relationships alongside data analysis.

As for definitions, *causal structures* are a class of tools to depict cause–effect relationships (defined and described in Grace et al., 2012). They include structural equation meta models (SEMMs), causal diagrams (CDs), and structural equation models (SEMs). At the highest level, SEMMs are conceptual networks that describe, at a broad theoretical level, relationships within a system. The nodes in these networks can include measurable and unmeasurable variables, concepts, and/or combinations of variables and are not tied to any taxonomic–geographical context but instead are generalizations of many observations. SEMMs have been effectively used to assimilate and evaluate competing theories about productivity–richness relationships in grasslands, for instance (Grace et al., 2016). At the intermediate level are CDs, which are more **specified** and serve to bridge the higher level theory to a specific study system of interest. They play a pivotal role in translating the theory described in an SEMM to the design and interpretation of a study and vice versa. CDs are the level at which most phylogeographical studies take place. At the most detailed level are SEMs, which are fully specified models that convey the precise variables measured in a study, their causal paths and the statistics used to quantify those paths. An SEM is a testable causal hypothesis for a given system whereas a causal diagram can be specified into different SEMs based on the design of a study. SEMs can be used to quantify the pathways proposed in a CD, and testing hierarchically nested SEMs can be used to determine the level of complexity necessary to describe a system (Grace et al., 2016). The application of SEMs to **Earth–life science** is a worthwhile topic that requires its own consideration and will not be discussed further here. A detailed review of causal structures and their implementation is found in Grace et al. (2012).

A starting point to defining causal structures for a system is to ask, “What variables are relevant?”. When drawing connections (edges) between variables (nodes) it becomes evident that some intermediary variables are missing if a parent variable does not have direct or complete causal influence on its child. A primary strength of causal analysis is its representation of system complexity in the form of compound paths (not just A → C, but A → B → C). This is due to the fact that causal analysis is based on a network graph (e.g., Figure 2b; Grace & Irvine, 2020). Using networks to represent hypotheses captures both **direct and indirect relationships** allowing for a more nuanced representation of reality.

Determining which variables are relevant in a system may be informed by applying a *chain of causal logic* in the form of two questions: (i) is there a known or conceivable **mechanism** through which *A* can affect *B*? and (ii) is *A* decomposable into other variables? For example, it can be argued that a mountain cannot directly control species distributions or divergence. It instead “acts” indirectly through its constituent direct variables, such as atmospheric oxygen concentration, temperature, solar insolation and precipitation (Table 1, Figure 4). These direct variables are easily measured, and importantly, they exert a measurable effect on an organism's biology through documented and/or quantifiable mechanisms (Table 2). For example, temperature is known to impact the energy invested in behavioural or physiological thermoregulation (e.g., finding shelter/shade, shivering, sweating), enzyme activity (Feller, 2010; Low et al., 1973; Peterson et al., 2007) and mutation rate (Berger et al., 2017; Garcia et al., 2010; Matsuba et al., 2012). These effects differ from those expected in response to an oxygen gradient, which instead include changes in oxygen–haemoglobin binding affinity (Miao et al., 2017) and haemoglobin concentration (Simonson et al., 2010). Still different patterns are expected from differential adaptation to UV burden, such as divergent responses within UV radiation receptor pathways (e.g., mediated by *UVR8*; Tossi et al., 2019) and the induction of protective phenolpropanoids in plants (Zeng et al., 2020). While observations of the richness or relatedness of populations in a geographical/geological context are important, more detailed assays into the physiology or the genome (e.g., to assess adaptation) may be necessary to answer many causal questions.

**TABLE 2:** Constituent (direct) variables are shown with the proposed intrinsic organismal effects they are thought to influence (right)

Constituent, direct variables	Intrinsic effect
Temperature	Growth rate
	Thermoregulation
	Enzyme efficiency
Insolation	Photosynthesis
	Mutation rate
	Growth rate
Precipitation	Osmoregulation
	Thermoregulation
*p*O_2_	Respiration
Daylight	Growth rate
	Reproductive timing

Note

This is not an exhaustive list and effects will vary by taxon.

Often not all variables can be evaluated in a study. However, drawing a causal diagram makes clear what variables *are* being tested, their presumed mode(s) of influence (causal pathways), which variables are excluded, and the presumed biological effect. As Dawson (2014) explained, studies can leverage naturally occurring combinations of variables on the landscape (“natural experiments,” *sensu* Dawson, 2014) to isolate individual effects similar to controlled laboratory experiments (Dawson, 2014; Gould & Johnston, 1972; Morris, 1995). Importantly, using causal structures to decompose aggregate features into direct variables should feed back to reveal something inherent about the features themselves. If we are interested in how important mountain building vs. river formation is to shaping biological evolution, it is reasonable to think that their relative power can be explained by the direct and indirect causal pathways each has to act on biology. Do aggregate features that are more influential have more causal pathways through which to work? If so, this could be a “rule” that describes a fundamental property of how earth processes shape life.

### 4.3 Applying causal structures

**FIGURE 6:**
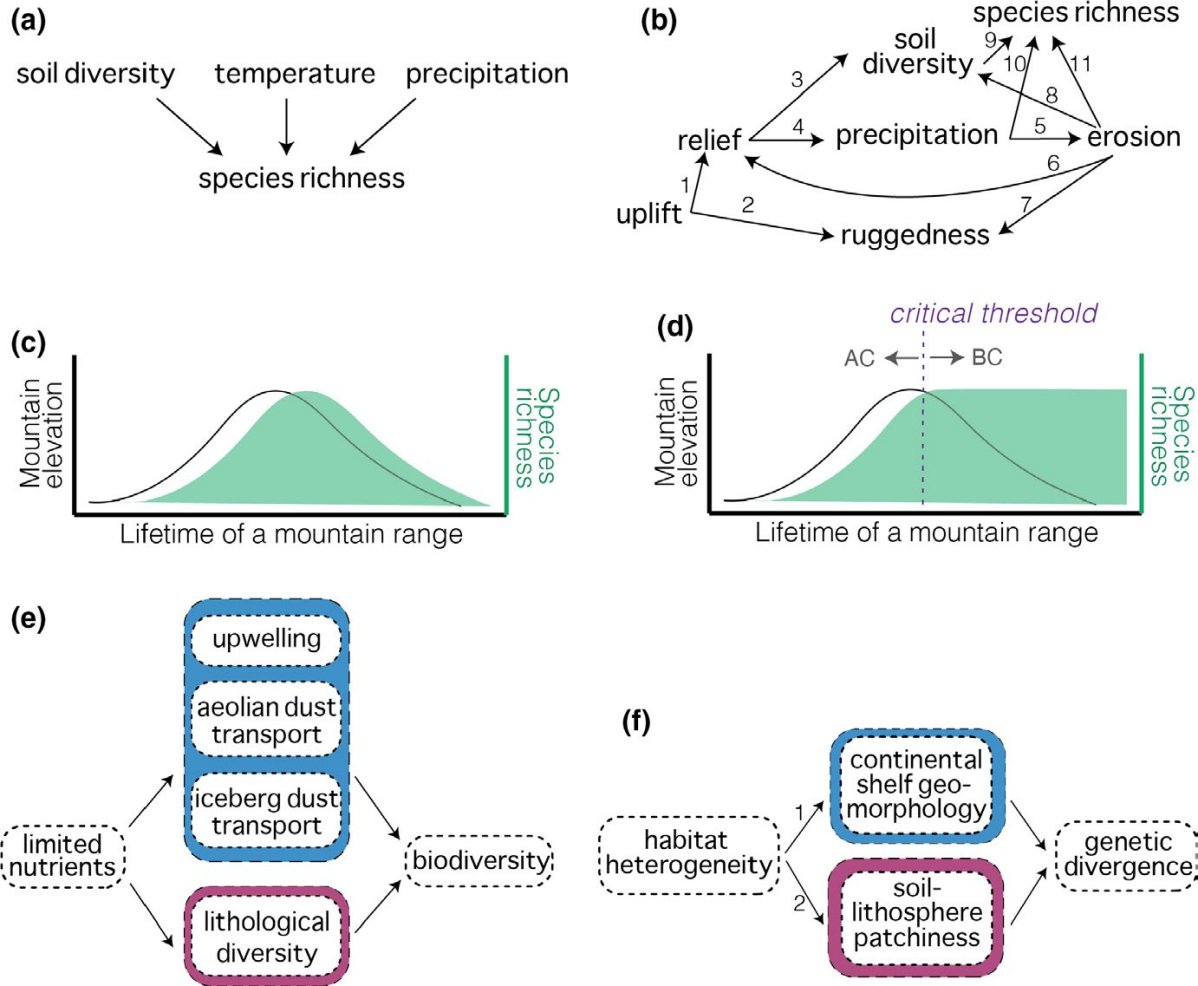
Examples of how causal structures can be used to convey knowledge or hypotheses about a system. (a) A causal diagram (CD) of the interpretations from Antonelli, Kissling, et al. (2018) regarding controls on species richness patterns in montane settings. (b) A detailed CD using geological knowledge to showcase how different processes would impact a hypothesized control (soil diversity) on species richness. Relationships are detailed in Table 3. Other relationships are possible with proper justification. Discussion of these variables and their paths are a natural point of discussion and collaboration across disciplines and study systems. (c) The expectation if species richness (green) depends on soil diversity and soil diversity is entirely abiotically generated. It would follow the birth, life, and death of montane topography. (d) Proposed expectation of species richness if a critical threshold is reached at which point soil formation switches from abiotic control (AC) to biotic control (BC) and is therefore retained following erosion of the topography. (e) A structural equation metamodel (SEMM) of how the lithological diversity (which generates soil diversity) hypothesized by Antonelli, Kissling, et al. (2018) is comparable to other abiotic processes that control nutrient fluxes. (f) An SEMM of how habitat heterogeneity (Rahbek, Borregaard, Antonelli, et al., 2019), fuelled by soil/lithosphere patchiness, could lead to genetic divergence through differential adaptation. This mechanism is comparable to population isolation due to the patchiness of marginal marine habitat caused by heterogeneous morphology of continental shelves (Dolby et al., 2020), which is expected to produce more nonadaptive divergence. Blue denotes marine processes and pink denotes terrestrial processes. Graph conventions follow Grace et al. (2012)

In this section we will show how SEMMs and CDs can be applied to Earth–life systems and what we can learn from their application. Over the past decade, mountain ranges have garnered tremendous attention as putative generators of biodiversity (Antonelli, Kissling, et al., 2018; Hoorn et al., 2010, 2013; Rahbek, Borregaard, Antonelli, et al., 2019). This comes from observations that many mountain ranges have high numbers of species, which suggests they either accumulate biodiversity or promote the origination of lineages *in situ*. This has led researchers to ask, “What is it *about* mountains that leads to high diversity?” According to our criteria, a mountain is an aggregate feature—it is decomposable into a suite of direct causal variables. Using our framework here we might more directly ask, “What are the *direct* causal controls on biodiversity within aggregate mountain systems?” Work by Antonelli, Kissling, et al. (2018) proposed the main controls on species richness in montane settings to be soil heterogeneity, temperature, and precipitation, which is depicted in a causal diagram in Figure 6a. Focusing on soil diversity, the authors proposed that soil diversity was due to **lithological** diversity. Others proposed that the entrainment, **uplift** and exposure of partially melted oceanic crust at subduction zones provides key nutrients or leads to the development of specific soil types that require specialized adaptation for organisms to inhabit (e.g., **serpentine soils**; Rahbek, Borregaard, Antonelli, et al., 2019). We can make a more detailed causal diagram for the controls on soil heterogeneity based on this hypothesis. We know that soil formation would depend on the rate of erosion and exposure of the bedrock, which involve several variables (Figure 6b) and the presumed causal relationships amongst these are described in Table 3. Drawing these diagrams teaches us two main lessons.

**TABLE 3:** Explanation of relationships used to justify pathways drawn in Figure 6

Path	Explanation of relationship
1	Uplift increases surface relief
2	Uplift of the surface is not uniform, leading to uneven (rugged) topography
3	Thickened lithosphere (relief) houses mineral/nutrient “reservoir”
4	Surface relief leads to adiabatic cooling, causing precipitation
5	Precipitation causes erosion through abrasion, attrition, shear stress, etc.
6	Erosion breaks down and removes rock mass, decreasing relief
7	Erosion (e.g., rivers) incises topography, increasing ruggedness
8	Erosion removes and cuts into lithosphere, revealing new surface area
9	Soil diversity controls nutrients available for biology
10	Precipitation controls water availability for biology
11	Erosion can form peaks and valleys that can isolate populations, leading to divergence, which increases species richness

Note

An alternative to path 11 can be proposed that instead connects ruggedness to species richness. Path 11 was proposed by Antonelli, Kissling, et al. (2018) but routing the pathway instead through ruggedness suggests that erosion does not directly affect species richness but does so indirectly.

The first lesson is that the system dynamics detailed in Figure 6b lead to several predictions. Prediction one is that there should be a correlation between soil diversity and species richness; indeed, Antonelli, Kissling, et al. (2018) showed this relationship, but it could be tested further, for example over different spatial scales. The second prediction is that mountains formed by subduction of oceanic crust should have higher richness than mountains at continent–continent collisions which have more silica‐based **lithology** with lower concentrations of iron‐ or magnesium‐bearing minerals. For example, all else being equal, the Himalaya should have lower richness than the Andes mountains; and this is also consistent with their findings (Antonelli, Kissling, et al., 2018; Rahbek, Borregaard, Antonelli, et al., 2019). The third prediction is that the variability of mineral composition of oceanic crust (e.g., Arevalo & McDonough, 2010) may manifest an effect on species richness; perhaps soils could be compared from hotspot‐driven ocean islands vs. subduction‐driven mountains to test this prediction. Lastly, but most importantly, erosion and **exhumation** rates along or between mountains should play a key role because lithosphere is the first rate‐limiting step of providing fresh material from which soils can form. The diagram helps us hypothesize that mountains with higher erosion rates due to faster uplift rates or greater precipitation might lead to faster soil generation and higher richness. Indeed, Antonelli, Kissling, et al. (2018) found an effect of erosion rate on richness, but more detailed study could better constrain this relationship in different regions, perhaps to decouple a signal of climate from a signal of uplift. The relationship could also be tested at different spatial scales.

From this logic, it also follows that because erosion rate is coupled to **uplift** rate, when uplift slows, the tectonically controlled rate of soil formation may decrease and therefore richness may also decrease. If this were true, we would expect a normal distribution of richness over time that mirrors the life of the mountain itself (Figure 6c). Alternatively, there could be a latency period in which growth of topography leads to abiotically driven soil formation, but after some time the biological community contributes to or becomes the main generator of soil. If so, the biotic system would have entered a self‐perpetuating state where nutrients are recycled by the biotic community and become decoupled from and are no longer controlled by the exhumation or erosion of the bedrock (Figure 6d). This second scenario implies: (i) the causal control on diversity shifts from abiotic to biotic at some critical threshold; and (ii) mountains “launch” biological diversity but diversity maintains diversity. These speculations would require explicit testing but offer predictions against which new observations can be compared (Figure 6c vs. d). In summary, lesson one is that casual structures can illuminate relationships and guide hypotheses in Earth–life systems. Some hypotheses may be testable in mountain systems, but some may be better tested in other settings or under controlled conditions, such as at biological field stations.

The second lesson of translating these results into causal diagrams transfers knowledge in the opposite direction. Instead of motivating new hypotheses, we can generalize the causal diagram in Figure 6a into an SEMM by leveraging knowledge from other studies. Most simply, the interpretation from Antonelli, Kissling, et al. (2018) boils down to the availability of nutrients and their spatial heterogeneity (patchiness). This result can be abstracted into an SEMM that includes additional observations from the literature. It is well documented that oceanic bottom waters become enriched in nutrients over time due to the deposition and decay of organic matter (Christian & Lewis, 1997). **Upwelling** of these nutrient‐rich bottom waters in coastal areas causes high biomass and diversity (Pauly & Christensen, 1995), such as in kelp forests off the western coast of the Americas (Winkler et al., 2017). Likewise, waters of the open ocean are often nutrient‐depleted and the blowing of aeolian dust from land and deposition of dust from icebergs into oligotrophic waters can bring trace elements, particularly iron, that fuels the patchy increase of biomass and productivity (if not diversity *per se*; Moore et al., 1984; Aumont et al., 2008; Raiswell et al., 2008; Maher et al., 2010). In essence, these are different examples of how abiotic processes control nutrient diversity/abundance, which in turn control species richness or biomass (Figure 6e). There are probably many more such examples. The abstraction of this knowledge into an SEMM shows that the soil diversity hypothesis posed for mountain regions fits within existing knowledge from marine ecosystems. They are conceptually linked, even though they are usually separated in practice. A second interpretation from Rahbek, Borregaard, Antonelli, et al. (2019) proposed that lithological heterogeneity could lead to local soil characteristics that require special biological adaptations to inhabit (e.g., serpentine soils). This could lead to speciation by differential adaptation, thereby increasing richness. Another SEMM (Figure 6f) contextualizes this idea to formalize patchiness of abiotic conditions as another phenomenon that links terrestrial and marine systems. In marginal marine environments, work has shown that the steepness of **continental shelves** can restrict and isolate habitat types, leading to isolation of habitat patches, population divergence and potentially high richness (Dolby et al., 2018, 2020) as well as demographic changes (Stiller et al., 2020). By drawing this SEMM we again see that patchiness of minerals (due to lithological heterogeneity) or patchiness of land steepness are conceptually related. The main difference is that pathway 1 only implies physical isolation, although differential adaptation is possible (Dolby, 2021), whereas the assumption of pathway 2 requires differential adaptation and would best be tested with genomic or common garden methods (Figure 6f). In this section I showed how we can translate the results of studies into causal diagrams, new predictions, and an expanded conceptualization of results that follows. Less obvious but equally important, these diagrams show what variables were not considered in these studies (and therefore my models), such as solar insolation, that could easily be tested in future studies. These structures could further be used to test other evolutionary or ecological hypotheses, such as how environmental factors control the evolution of morphology across species (Madden, 2014). Alternatively, causal structures could be used to integrate complementary palaeontological and molecular data to develop more holistic and integrated models of diversity gradients or species evolution (Badgley et al., 2017).

I hope I have shown that a mountain is many things. If abiotic factors can be accounted for causally, then it would reveal to what degree diversity and diversification patterns are only explainable through the processes of biology itself. One could imagine extending the causal structures drawn here to include biological feedbacks and complexities, leading to a network that integrates abiotic and biotic components to holistically describe the mountain–life system. It could even be temporally explicit! If there are thresholds whereby abiotic processes foster diversification or richness that becomes self‐perpetuating, then the original abiotic control structure would splinter at some tipping point and shift causal control to the biology. In essence, biology would come under the control of its own causal schema. Since we know that at some point long ago life originated from nonlife, perhaps such causal transitions are not so strange. Perhaps they are even a hallmark of the Earth–life system. Employing these structures and an understanding of causal systems may be a way to formally bridge ecology, evolution and geology. More work is needed.

## 5 CONCLUSIONS

Top‐down causation is proposed to have widely shaped the history of life on Earth (Davies, 2011; Walker & Davies, 2012). Questions about how earth processes shape the diversification and distribution of life are fundamentally questions about how to describe causal relationships within the Earth–life system. Here, I have proposed that at the species scale, earth processes can be considered to have a quasideterministic effect on biology. These earth processes are often co‐occurring and potentially pseudocongruent, and the landscape features they form are combinations of direct, quantifiable variables. These two factors motivate the need for developing more refined ways of articulating and testing causal hypotheses that facilitate interdisciplinary research. I demonstrate how to do this using causal structures, specifically SEMMs and CDs. Their application here suggests new tests to characterize the relationship between lithological diversity and species richness, as well as recontextualizing knowledge into theory about how earth processes redistribute nutrients that control biomass, and cause population isolation through patchiness that can lead to speciation. Interrogating these causal relationships led to speculation that some Earth–life systems may encounter a redistribution of causal control from abiotic to biotic, suggesting temporal dynamics may be relevant. Finally, these tools are broadly applicable and will help develop more mechanistic knowledge while helping to bridge geology, evolution and ecology. I hope they will help us better answer higher‐order questions about what earth processes most generate new species, and how and under what limits they operate over spatial, temporal and taxonomic dimensions.

## 6 Glossary

**Bathymetry:** Water depth in bodies of water (e.g., oceans, lakes). **Collinear:** Within statistics, when multiple predictor variables are correlated. **Continental shelves:** The shallow underwater area surrounding landmasses. **Direct relationship:** Within graph theory, when one variable has a single pathway leading to another variable (A C). **Directed acyclic graph:** Within graph theory, a network composed of nodes and edges in which there are no directed cycles (i.e., no closed loops). **Exhumation:** Within geology, the exposure of new rock to the atmosphere. **Earth–life sciences:** Any research focus that deeply integrates the earth sciences and the life sciences. **Epiphenomenon:** A phenomenon that is correlated with or a byproduct of the phenomenon of interest. **Incision:** Within geology, the erosional process of a river cutting a path into rock. **Indirect relationship:** Within graph theory, when one variable has a compound pathway to a second variable that goes through an intermediate variable (A B C). **Lithology:** Within geology, the type or characteristics of rocks. **Mechanism:** The detailed mode or process by which an observed phenomenon comes to be. **Monsoon:** An organized, regional climate pattern that changes seasonally, bringing changes in precipitation. **Precession:** Within geology, a Milankovitch cycle describing how the tilt of the Earth’s axis changes over ~23,000‐year cycles (i.e. Earth’s wobble around its own axis), which changes global solar insolation patterns. **Pseudocongruence:** The phenomenon whereby more than one process can produce a similar effect. **Serpentine soils:** Soil derived from ultramafic (low silica‐bearing) rocks, generally high in magnesium and low in calcium and nitrogen. **Solar insolation:** The solar radiation, measured as the power per unit area, that is received from the sun. **Specification:** Within statistics, the process of building a statistical model that includes the assignment of data to variables and the statistical framework used. **Uplift:** Within geology, the vertical upwards movement of Earth’s surface. **Upwelling:** Within geology, the process by which cold, deep waters rise to the ocean’s surface.

## AUTHOR CONTRIBUTIONS

G.A.D. conceived of, wrote and revised this manuscript.

## Data Availability

No data were generated for or used in this paper.

## References

[mec16142-bib-0001] Antonelli, A. , Ariza, M. , Albert, J. , Andermann, T. , Azevedo, J. , Bacon, C. , Faurby, S. , Guedes, T. , Hoorn, C. , Lohmann, L. G. , Matos‐Maraví, P. , Ritter, C. D. , Sanmartín, I. , Silvestro, D. , Tejedor, M. , ter Steege, H. , Tuomisto, H. , Werneck, F. P. , Zizka, A. , & Edwards, S. V. (2018). Conceptual and empirical advances in Neotropical biodiversity research. PeerJ, 6, e5644–e5653. 10.7717/peerj.5644 30310740 PMC6174874

[mec16142-bib-0002] Antonelli, A. , Kissling, W. D. , Flantua, S. G. A. , Bermúdez, M. A. , Mulch, A. , Muellner‐Riehl, A. N. , Kreft, H. , Linder, H. P. , Badgley, C. , Fjeldså, J. , Fritz, S. A. , Rahbek, C. , Herman, F. , Hooghiemstra, H. , & Hoorn, C. (2018). Geological and climatic influences on mountain biodiversity. Nature Geoscience, 11, 718–726. 10.1038/s41561-018-0236-z

[mec16142-bib-0003] Arevalo, R. Jr , & McDonough, W. F. (2010). Chemical variations and regional diversity observed in MORB. Chemical Geology, 271, 70–85. 10.1016/j.chemgeo.2009.12.013

[mec16142-bib-0004] Aumont, O. , Bopp, L. , & Schulz, M. (2008). What does temporal variability in aeolian dust deposition contribute to sea‐surface iron and chlorophyll distributions? Geophysical Research Letters, 35, L07607. 10.1029/2007GL031131

[mec16142-bib-0005] Badgley, C. , Smiley, T. M. , Terry, R. , Davis, E. B. , DeSantis, L. R. G. , Fox, D. L. , Hopkins, S. S. B. , Jezkova, T. , Matocq, M. D. , Matzke, N. , McGuire, J. L. , Mulch, A. , Riddle, B. R. , Roth, V. L. , Samuels, J. X. , Strömberg, C. A. E. , & Yanites, B. J. (2017). Biodiversity and topographic complexity: modern and geohistorical perspectives. TREE, 32, 211–226. 10.1016/j.tree.2016.12.010 28196688 PMC5895180

[mec16142-bib-0006] Balao, F. , Navarro‐Sampedro, L. , Berjano, R. , García‐Castaño, J. L. , Casimiro‐Soriguer, R. , Talavera, M. , Talavera, S. , & Terrab, A. (2017). Riverine speciation and long dispersal colonization in the Ibero‐African *Onopordum dissectum* complex (Asteraceae). Biological Journal of the Linnean Society, 183, 600–615. 10.1093/botlinnean/bow022

[mec16142-bib-0007] Berger, D. , Stångberg, J. , Grieshop, K. , Martinossi‐Allibert, I. , & Arnqvist, G. (2017). Temperature effects on life‐history trade‐offs, germline maintenance and mutation rate under simulated climate warming. Proceedings of the Royal Society B: Biological Sciences, 284, 20171721–20171810. 10.1098/rspb.2017.1721 PMC5698646 29118134

[mec16142-bib-0008] Blount, Z. D. , Lenski, R. E. , & Losos, J. B. (2018). Contingency and determinism in evolution: Replaying life’s tape. Science, 362, 5979–6012. 10.1126/science.aam5979 30409860

[mec16142-bib-0009] Brockerville, R. M. , McGrath, M. J. , Pilgrim, B. L. , & Marshall, H. D. (2013). Sequence analysis of three pigmentation genes in the Newfoundland population of Canis latrans links the Golden Retriever Mc1r variant to white coat color in coyotes. Mammalian Genome: Official Journal of the International Mammalian Genome Society, 24, 134–141. 10.1007/s00335-012-9443-x 23297074

[mec16142-bib-0010] Brozovi, N. , Burbank, D. , & Meigs, A. (1997). Climatic Limits on Landscape Development in the Northwestern Himalaya. Science, 276, 571–574. 10.1126/science.276.5312.571 9110972

[mec16142-bib-0011] Champagnac, J.‐D. , Molnar, P. , Sue, C. , & Herman, F. (2012). Tectonics, climate, and mountain topography. Journal of Geophysical Research: Atmospheres, 117(B02403), 1–34.

[mec16142-bib-0012] Chapman, M. A. , Hiscock, S. J. , & Filatov, D. A. (2013). Genomic divergence during speciation driven by adaptation to altitude. Molecular Biology and Evolution, 30, 2553–2567. 10.1093/molbev/mst168 24077768 PMC3840311

[mec16142-bib-0013] Christian, J. R. , Lewis, M. R. , & Karl, D. M. (1997). Vertical fluxes of carbon, nitrogen, and phosphorus in the North Pacific Subtropical Gyre near Hawaii. Journal of Geophysical Research Letters, 102, 15667–15677. 10.1029/97JC00369

[mec16142-bib-0014] Colosimo, P. F. (2005). Widespread parallel evolution in sticklebacks by repeated fixation of ectodysplasin alleles. Science, 307, 1928–1933. 10.1126/science.1107239 15790847

[mec16142-bib-0015] Colwell, R. K. , & Hurtt, G. C. (1994). Nonbiological gradients in species richness and a spurious Rapoport effect. The American Naturalist, 144, 570–595. 10.1086/285695

[mec16142-bib-0016] Coyne, J. A. , & Orr, H. A. (2004). Speciation. Sinauer Associates.

[mec16142-bib-0017] Crandall, E. D. , Riginos, C. , Bird, C. E. , Liggins, L. , Treml, E. , Beger, M. , Barber, P. H. , Connolly, S. R. , Cowman, P. F. , DiBattista, J. D. , Eble, J. A. , Magnuson, S. F. , Horne, J. B. , Kochzius, M. , Lessios, H. A. , Liu, S. Y. V. , Ludt, W. B. , Madduppa, H. , Pandolfi, J. M. , … Gaither, M. R. (2019). The molecular biogeography of the Indo‐Pacific: Testing hypotheses with multispecies genetic patterns. Global Ecology and Biogeography, 28, 943–960. 10.1111/geb.12905

[mec16142-bib-0018] Davies, P. C. W. (2011). The epigenome and top‐down causation. Interface Focus, 2, 42–48. 10.1098/rsfs.2011.0070 22419988 PMC3262298

[mec16142-bib-0019] Dawson, M. N. (2014). Natural experiments and meta‐analyses in comparative phylogeography. Journal of Biogeography, 41, 52–65. 10.1111/jbi.12190

[mec16142-bib-0020] Deng, J. , Auchtung, J. M. , Konstantinidis, K. T. et al (2019). Genomic variations underlying speciation and niche specialization of *Shewanella baltica* . mSystems, 4. 10.1128/mSystems.00560-19 PMC6794122 31615877

[mec16142-bib-0021] Dielforder, A. , Hetzel, R. , & Oncken, O. (2020). Megathrust shear force controls mountain height at convergent plate margins. Nature, 582, 225–229. 10.1038/s41586-020-2340-7 32528094

[mec16142-bib-0022] Dolby, G. A. (2021). The hidden landscape: Evidence that sea‐level change shaped the present population genomic patterns of marginal marine species. Molecular Ecology, 30, 1357–1360. 10.1111/mec.15826 33545743

[mec16142-bib-0023] Dolby, G. A. , Bedolla, A. M. , Bennett, S. E. , & Jacobs, D. K. (2020). Global physical controls on estuarine habitat distribution during sea level change_ Consequences for genetic diversification through time. Global and Planetary Change, 187, 103128.

[mec16142-bib-0024] Dolby, G. A. , Bennett, S. E. , Lira‐Noriega, A. , Wilder, B. T. , & Munguía‐Vega, A. (2015). Assessing the geological and climatic forcing of biodiversity and evolution surrounding the Gulf of California. Journal of the Southwest, 57, 391–455. 10.1353/jsw.2015.0005

[mec16142-bib-0025] Dolby, G. A. , Dorsey, R. J. , & Graham, M. R. (2019). A legacy of geo‐climatic complexity and genetic divergence along the lower Colorado River: Insights from the geological record and 33 desert‐adapted animals. Journal of Biogeography, 46, 2479–2505. 10.1111/jbi.13685

[mec16142-bib-0026] Dolby, G. A. , Ellingson, R. A. , Findley, L. T. , & Jacobs, D. K. (2018). How sea level change mediates genetic divergence in coastal species across regions with varying tectonic and sediment processes. Molecular Ecology, 27, 994–1011. 10.1111/mec.14487 29336083

[mec16142-bib-0027] Edwards, T. , Tollis, M. , Hsieh, P. et al (2016). Assessing models of speciation under different biogeographic scenarios; an empirical study using multi‐locus and RNA‐seq analyses. Ecology and Evolution, 102, 1–18. 10.1002/ece3.1865 PMC4729248 26843925

[mec16142-bib-0028] Egholm, D. L. , Nielsen, S. B. , Pedersen, V. K. , & Lesemann, J. E. (2009). Glacial effects limiting mountain height. Nature, 460, 884–887. 10.1038/nature08263 19675651

[mec16142-bib-0029] Eisenhauer, N. , Bowker, M. A. , Grace, J. B. , & Powell, J. R. (2015). From patterns to causal understanding: Structural equation modeling (SEM) in soil ecology. Pedobiologia ‐ Journal of Soil Ecology, 58, 65–72. 10.1016/j.pedobi.2015.03.002

[mec16142-bib-0030] Favre, A. , Widmer, A. , & Karrenberg, S. (2017). Differential adaptation drives ecological speciation in campions (Silene): evidence from a multi‐site transplant experiment. The New Phytologist, 213, 1487–1499. 27775172 10.1111/nph.14202

[mec16142-bib-0031] Feldman, C. R. , & Spicer, G. S. (2006). Comparative phylogeography of woodland reptiles in California: repeated patterns of cladogenesis and population expansion. Molecular Ecology, 15, 2201–2222. 10.1111/j.1365-294X.2006.02930.x 16780435

[mec16142-bib-0032] Feller, G. (2010). Protein stability and enzyme activity at extreme biological temperatures. Journal of Physics: Condensed Matter, 22, 323101–323118. 10.1088/0953-8984/22/32/323101 21386475

[mec16142-bib-0033] Flessa, K. W. , & Levinton, J. S. (1975). Phanerozoic diversity patterns: Tests for randomness. The Journal of Geology, 83, 239–248. 10.1086/628084

[mec16142-bib-0034] Ford, A. G. P. , Rüber, L. , Newton, J. , Dasmahapatra, K. K. , Balarin, J. D. , Bruun, K. , & Day, J. J. (2016). Niche divergence facilitated by fine‐scale ecological partitioning in a recent cichlid fish adaptive radiation. Evolution, 70, 2718–2735. 10.1111/evo.13072 27659769 PMC5132037

[mec16142-bib-0035] Garcia, A. M. , Calder, R. B. , Dollé, M. E. T. , Lundell, M. , Kapahi, P. , & Vijg, J. (2010). Age‐ and Temperature‐Dependent Somatic Mutation Accumulation in Drosophila melanogaster. PLoS Genetics, 6, e1000950–e1000958. 10.1371/journal.pgen.1000950 20485564 PMC2869313

[mec16142-bib-0036] Gharnit, E. , Bergeron, P. , Garant, D. , & Realea, D. (2020). Exploration profiles drive activity patterns and temporal niche specialization in a wild rodent. Behavioral Ecology, 31, 772–783. 10.1093/beheco/araa022

[mec16142-bib-0037] Gould, S. J. (1989). Wonderful life. W. W. Norton & Company.

[mec16142-bib-0038] Gould, S. J. (1994). The evolution of life on the Earth. Scientific American, 271, 84–91. 10.1038/scientificamerican1094-84 7939569

[mec16142-bib-0039] Gould, S. J. , & Johnston, R. F. (1972). Geographic variation. Annual Review of Ecology, Evolution, and Systematics, 3, 457–498. 10.1146/annurev.es.03.110172.002325

[mec16142-bib-0040] Grace, J. B. (2006). Structural equation modeling and natural systems. Cambridge University Press.

[mec16142-bib-0041] Grace, J. B. (2010). Structural equation modeling for observational studies. The Journal of Wildlife Management, 72, 14–22. 10.2193/2007-307

[mec16142-bib-0042] Grace, J. B. (2015). Taking a systems approach to ecological systems. Journal of Vegetation Science, 26, 1025–1027. 10.1111/jvs.12340

[mec16142-bib-0043] Grace, J. B. , Anderson, T. M. , Olff, H. , & Scheiner, S. M. (2010). On the specification of structural equation models for ecological systems. Ecological Monographs, 80, 67–87. 10.1890/09-0464.1

[mec16142-bib-0044] Grace, J. B. , Anderson, T. M. , Seabloom, E. W. , Borer, E. T. , Adler, P. B. , Harpole, W. S. , Hautier, Y. , Hillebrand, H. , Lind, E. M. , Pärtel, M. , Bakker, J. D. , Buckley, Y. M. , Crawley, M. J. , Damschen, E. I. , Davies, K. F. , Fay, P. A. , Firn, J. , Gruner, D. S. , Hector, A. , … Smith, M. D. (2016). Integrative modelling reveals mechanisms linking productivity and plant species richness. Nature, 529, 390–393. 10.1038/nature16524 26760203

[mec16142-bib-0045] Grace, J. B. , & Bollen, K. A. (2007). Representing general theoretical concepts in structural equation models: the role of composite variables. Environmental and Ecological Statistics, 15, 191–213.

[mec16142-bib-0046] Grace, J. B. , & Irvine, K. M. (2020). Scientist’s guide to developing explanatory statistical models using causal analysis principles. Ecology, 101, 441–515. 10.1002/ecy.2962 31872426

[mec16142-bib-0047] Grace, J. B. , Schoolmaster, D. R. , Guntenspergen, G. R. , Little, A. M. , Mitchell, B. R. , Miller, K. M. , & Schweiger, E. W. (2012). Guidelines for a graph‐theoretic implementation of structural equation modeling. Ecosphere, 3, 73–45. 10.1890/ES12-00048.1

[mec16142-bib-0048] Gunderson, A. R. , Mahler, D. L. , & Leal, M. (2018). Thermal niche evolution across replicated Anolis lizard adaptive radiations. Proceedings of the Royal Society B: Biological Sciences, 285. 10.1098/rspb.2017.2241 PMC5936720 29669895

[mec16142-bib-0049] Hickerson, M. J. (2014). All models are wrong. Molecular Ecology, 23, 2887–2889. 10.1111/mec.12794 24931159

[mec16142-bib-0050] Hodkinson, I. D. (2005). Terrestrial insects along elevation gradients: species and community responses to altitude. Biological Reviews, 80, 489–525. 10.1017/S1464793105006767 16094810

[mec16142-bib-0051] Hoorn, C. , Mosbrugger, V. , Mulch, A. , & Antonelli, A. (2013). Biodiversity from mountain building. Nature Geoscience, 6, 154. 10.1038/ngeo1742

[mec16142-bib-0052] Hoorn, C. , Wesselingh, F. P. , ter Steege, H. , Bermudez, M. A. , Mora, A. , Sevink, J. , Sanmartin, I. , Sanchez‐Meseguer, A. , Anderson, C. L. , Figueiredo, J. P. , Jaramillo, C. , Riff, D. , Negri, F. R. , Hooghiemstra, H. , Lundberg, J. , Stadler, T. , Sarkinen, T. , & Antonelli, A. (2010). Amazonia through time: andean uplift, climate change, landscape evolution, and biodiversity. Science, 330, 927–931. 10.1126/science.1194585 21071659

[mec16142-bib-0053] Hopkins, M. , & Pearl, J. (2007). Causality and counterfactuals in the situation calculus. Journal of Logic and Computation, 17, 939–953. 10.1093/logcom/exm048

[mec16142-bib-0054] Hudson, E. J. , & Price, T. D. (2014). Pervasive reinforcement and the role of sexual selection in biological speciation. The Journal of Heredity, 105, 821–833. 10.1093/jhered/esu041 25149257

[mec16142-bib-0055] Kolata, G. B. (1975). Paleobiology: Random events over geological time. Science, 189, 625–660. 10.1126/science.189.4203.625 17838750

[mec16142-bib-0056] Lapointe, F. J. , & Rissler, L. J. (2005). Congruence, consensus, and the comparative phylogeography of codistributed species in California. The American Naturalist, 166, 290–299. 10.1086/431283 16032580

[mec16142-bib-0057] Leaché, A. D. , Oaks, J. R. , Ofori‐Boateng, C. , & Fujita, M. K. (2020). Comparative phylogeography of West African amphibians and reptiles. Evolution, 74, 716–724. 10.1111/evo.13941 32067219

[mec16142-bib-0058] Li, R. , Tsaih, S.‐W. , Shockley, K. , Stylianou, I. M. , Wergedal, J. , Paigen, B. , & Churchill, G. A. (2006). Structural model analysis of multiple quantitative traits. PLoS Genetics, 2, 1046–1057. 10.1371/journal.pgen.0020114 PMC1513264 16848643

[mec16142-bib-0059] Losos, J. B. , Leal, M. , Glor, R. E. , de Queiroz, K. , Hertz, P. E. , Rodríguez Schettino, L. , Chamizo Lara, A. , Jackman, T. R. , & Larson, A. (2003). Niche lability in the evolution of a Caribbean lizard community. Nature, 424, 542–545. 10.1038/nature01814 12891355

[mec16142-bib-0060] Low, P. S. , Bada, J. L. , & Somero, G. N. (1973). Temperature adaptation of enzymes: roles of the free energy, the enthalpy, and the entropy of activation. Proceedings of the National Academy of Sciences of the USA, 70, 430–432. 10.1073/pnas.70.2.430 4510286 PMC433275

[mec16142-bib-0061] Lugon‐Moulin, N. , Brünner, H. , Balloux, F. , Hausser, J. , & Goudet, J. (1999). Do riverine barriers, history or introgression shape the genetic structuring of a common shrew (*Sorex araneus*) population? Heredity, 83, 155–161. 10.1046/j.1365-2540.1999.00567.x 10469203

[mec16142-bib-0062] Madden, R. H. (2014). Hypsodonty in Mammals: Evolution, geomorphology, and the role of Earth surface processes. University of Chicago Press.

[mec16142-bib-0063] Maher, B. A. , Prospero, J. M. , Mackie, D. , Gaiero, D. , Hesse, P. P. , & Balkanski, Y. (2010). Global connections between aeolian dust, climate and ocean biogeochemistry at the present day and at the last glacial maximum. Earth Science Reviews, 99, 61–97. 10.1016/j.earscirev.2009.12.001

[mec16142-bib-0064] Martin, M. D. , & Mendelson, T. C. (2016). The accumulation of reproductive isolation in early stages of divergence supports a role for sexual selection. Journal of Evolutionary Biology, 29, 676–689. 10.1111/jeb.12819 26717252

[mec16142-bib-0065] Matsuba, C. , Ostrow, D. G. , Salomon, M. P. , Tolani, A. , & Baer, C. F. (2012). Temperature, stress and spontaneous mutation in *Caenorhabditis briggsae* and *Caenorhabditis elegans* . Biology Letters, 9, 20120334. 22875817 10.1098/rsbl.2012.0334 PMC3565477

[mec16142-bib-0066] McClain, C. R. , & Etter, R. J. (2005). Mid‐domain models as predictors of species diversity patterns: Bathymetric diversity gradients in the deep sea. Oikos, 109, 555–566. 10.1111/j.0030-1299.2005.13529.x

[mec16142-bib-0067] Miao, B. , Wang, Z. , & Li, Y. (2017). Genomic analysis reveals hypoxia adaptation in the Tibetan mastiff by introgression of the gray wolf from the Tibetan Plateau. Molecular Biology and Evolution, 34, 734–743. 27927792 10.1093/molbev/msw274

[mec16142-bib-0068] Moore, R. M. , Milley, J. E. , & Chatt, A. (1984). The potential for biological mobilization of trace‐elements from aeolian dust in the ocean and its importance in the case of iron. Oceanologica Acta, 7, 221–228.

[mec16142-bib-0069] Morris, S. C. (1995). Ecology in deep time. Trends in Ecology & Evolution, 10, 290–294. 10.1016/S0169-5347(00)89104-1 21237046

[mec16142-bib-0070] Morris, S. C. (2006). Darwin's dilemma: the realities of the Cambrian ‘explosion’. Philosophical Transactions of the Royal Society B: Biological Sciences, 361, 1069–1083. 10.1098/rstb.2006.1846 PMC1578734 16754615

[mec16142-bib-0071] Morris, S. C. (2010). Evolution: like any other science it is predictable. Philosophical Transactions of the Royal Society B: Biological Sciences, 365, 133–145. 10.1098/rstb.2009.0154 PMC2842699 20008391

[mec16142-bib-0072] Mundy, N. I. (2005). A window on the genetics of evolution: MC1R and plumage colouration in birds. Proceedings of the Royal Society B: Biological Sciences, 272, 1633–1640. 10.1098/rspb.2005.3107 PMC1559852 16087416

[mec16142-bib-0073] Myers, E. A. , Hickerson, M. J. , & Burbrink, F. T. (2016). Asynchronous diversification of snakes in the North American warm deserts. Journal of Biogeography, 44, 461–474. 10.1111/jbi.12873

[mec16142-bib-0074] Naka, L. N. , & Brumfield, R. T. (2018). The dual role of Amazonian rivers in the generation and maintenance of avian diversity. Science. Advances, 4, eaar8575. 30083603 10.1126/sciadv.aar8575 PMC6070317

[mec16142-bib-0075] Ornelas, J. F. , García, J. M. , Ortiz‐Rodriguez, A. E. et al (2018). Tracking host trees: the phylogeography of endemic *Psittacanthus sonorae* (Loranthaceae) mistletoe in the Sonoran Desert. Journal of Heredity, 110, 229–246. 10.1093/jhered/esy065 30496534

[mec16142-bib-0076] Otsuka, J. (2014). Using causal models to integrate proximate and ultimate causation. Biology & Philosophy, 30, 19–37. 10.1007/s10539-014-9448-9

[mec16142-bib-0077] Pauly, D. , & Christensen, V. (1995). Primary production required to sustain global fisheries. Nature, 374, 255–257. 10.1038/374255a0

[mec16142-bib-0078] Pearl, J. (1995). Causal diagrams for empirical research ‐ Discussion. Biometrika, 82, 702–710. 10.1093/biomet/82.4.702

[mec16142-bib-0079] Pearl, J. (1998). Graphical models for probabilistic and causal reasoning. In Quantified representation of uncertainty and imprecision (pp. 367–389). Chapman and Hall/CRC.

[mec16142-bib-0080] Pearl, J. (2009). Causal inference in statistics: An overview. Statistics Surveys, 3, 96–146. 10.1214/09-SS057

[mec16142-bib-0081] Pearl, J. (2019). The seven tools of causal inference, with reflections on machine learning. Communications of the ACM, 62, 54–60. 10.1145/3241036

[mec16142-bib-0082] Pearl, J. , & Verma, T. S. (1995). A theory of inferred causation. Studies in Logic and the Foundations of Mathematics, 134, 789–811.

[mec16142-bib-0083] Peres, C. A. , Patton, J. L. , & da Silva, M. N. (1996). Riverine barriers and gene flow in Amazonian saddle‐back tamarins. Folia Primatologica; International Journal of Primatology, 67, 113–124. 10.1159/000157213 9032947

[mec16142-bib-0084] Peterman, W. E. , & Pope, N. S. (2021). The use and misuse of regression models in landscape genetic analyses. Molecular Ecology, 30, 37–47. 10.1111/mec.15716 33128830

[mec16142-bib-0085] Peterson, M. E. , Daniel, R. M. , Danson, M. J. , & Eisenthal, R. (2007). The dependence of enzyme activity on temperature: determination and validation of parameters. Biochemical Journal, 402, 331–337. 10.1042/BJ20061143 17092210 PMC1798444

[mec16142-bib-0086] Powell, R. , & Mariscal, C. (2015). Convergent evolution as natural experiment: the tape of life reconsidered. Interface Focus, 5, 20150040. 10.1098/rsfs.2015.0040 26640647 PMC4633857

[mec16142-bib-0087] Pugesek, B. H. , & Grace, J. B. (1998). On the utility of path modelling for ecological and evolutionary studies. Functional Ecology, 12, 853–856.

[mec16142-bib-0088] Rabosky, D. L. , Chang, J. , Title, P. O. , Cowman, P. F. , Sallan, L. , Friedman, M. , Kaschner, K. , Garilao, C. , Near, T. J. , Coll, M. , & Alfaro, M. E. (2018). An inverse latitudinal gradient in speciation rate for marine fishes. Nature, 559, 1–20. 10.1038/s41586-018-0273-1 29973726

[mec16142-bib-0089] Rahbek, C. , Borregaard, M. K. , Antonelli, A. , Colwell, R. K. , Holt, B. G. , Nogues‐Bravo, D. , Rasmussen, C. M. Ø. , Richardson, K. , Rosing, M. T. , Whittaker, R. J. , & Fjeldså, J. (2019). Building mountain biodiversity: Geological and evolutionary processes. Science, 365, 1114–1119. 10.1126/science.aax0151 31515384

[mec16142-bib-0090] Rahbek, C. , Borregaard, M. K. , Colwell, R. K. , Dalsgaard, B. O. , Holt, B. G. , Morueta‐Holme, N. , Nogues‐Bravo, D. , Whittaker, R. J. , & Fjeldså, J. (2019). Humboldt's enigma: What causes global patterns of mountain biodiversity? Science, 365, 1108–1113. 10.1126/science.aax0149 31515383

[mec16142-bib-0091] Raiswell, R. , Benning, L. G. , Tranter, M. , & Tulaczyk, S. (2008). Bioavailable iron in the Southern Ocean: the significance of the iceberg conveyor belt. Geochemical Transactions, 9, 7–9. 10.1186/1467-4866-9-7 18513396 PMC2440735

[mec16142-bib-0092] Raup, D. M. , Gould, S. J. , Gould, S. J. , Schopf, T. J. M. , & Simberloff, D. S. (1973). Stochastic models of phylogeny and the evolution of diversity. The Journal of Geology, 81, 525–542. 10.1086/627905

[mec16142-bib-0093] Riddle, B. R. , & Hafner, D. J. (2006). A step‐wise approach to integrating phylogeographic and phylogenetic biogeographic perspectives on the history of a core North American warm deserts biota. Journal of Arid Environments, 66, 435–461. 10.1016/j.jaridenv.2006.01.014

[mec16142-bib-0094] Ritland, K. , Newton, C. , & Marshall, H. D. (2001). Inheritance and population structure of the white‐phased “Kermode” black bear. Current Biology, 11, 1468–1472. 10.1016/S0960-9822(01)00448-1 11566108

[mec16142-bib-0095] Scheiner, S. M. , Mitchell, R. J. , & Callahan, H. S. (2000). Using path analysis to measure natural selection. Journal of Evolutionary Biology, 13, 423–433. 10.1046/j.1420-9101.2000.00191.x

[mec16142-bib-0096] Schluter, D. , Clifford, E. A. , Nemethy, M. , & McKinnon, J. S. (2004). Parallel evolution and inheritance of quantitative traits. The American Naturalist, 163, 809–822. 10.1086/383621 15266380

[mec16142-bib-0097] Schopf, T. , Raup, D. M. , Gould, S. J. , & Simberloff, D. S. (1975). Genomic versus morphologic rates of evolution: influence of morphologic complexity. Paleobiology, 1, 63–70. 10.1017/S0094837300002207

[mec16142-bib-0098] Servedio, M. R. , & Boughman, J. W. (2017). The role of sexual selection in local adaptation and speciation. Annual Reviews, 48, 85–109.

[mec16142-bib-0099] Simonson, T. S. , Yang, Y. , Huff, C. D. , Yun, H. , Qin, G. , Witherspoon, D. J. , Bai, Z. , Lorenzo, F. R. , Xing, J. , Jorde, L. B. , Prchal, J. T. , & Ge, R. (2010). Genetic evidence for high‐altitude adaptation in Tibet. Science, 329, 72–75. 10.1126/science.1189406 20466884

[mec16142-bib-0100] Smith, E. , & Morowitz, H. J. (2016). The origin and nature of life on earth: The emergence of the fourth geosphere. Cambridge University Press.

[mec16142-bib-0101] Soltis, D. E. , Morris, A. B. , McLachlan, J. S. , Manos, P. S. , & Soltis, P. S. (2006). Comparative phylogeography of unglaciated eastern North America. Molecular Ecology, 15, 4261–4293. 10.1111/j.1365-294X.2006.03061.x 17107465

[mec16142-bib-0102] Steiner, C. C. , Weber, J. N. , & Hoekstra, H. E. (2007). Adaptive variation in beach mice produced by two interacting pigmentation genes. PLoS Biology, 5, e219. 10.1371/journal.pbio.0050219 17696646 PMC1945039

[mec16142-bib-0103] Stevens, G. C. (1989). The latitudinal gradient in geographical range: how so many species coexist in the tropics. The American Naturalist, 133, 240–256. 10.1086/284913

[mec16142-bib-0104] Stiller, J. , Fonseca, R. R. , Alfaro, M. E. et al (2020). Using ultraconserved elements to track the influence of sea‐level change on leafy seadragon populations. Molecular Ecology, 2, 688–717. 10.1111/mec.15744 33217068

[mec16142-bib-0105] Stocker, T. F. , Qin, D. , Plattner, G.‐K. , Alexander, L. V. , Allen, S. K. , Bindoff, N. L. , Bréon, F.‐M. , Church, J. A. , Cubasch, U. , Emori, S. , Forster, P. , Friedlingstein, P. , Gillett, N. , Gregory, J. M. , Hartmann, D. L. , Jansen, E. , Kirtman, B. , Knutti, R. , Krishna Kumar, K. , … Xie, S.‐P. (2013). Technical Sum‐ mary. In T. F. Stocker , D. Qin , G.‐K. Plattner , M. Tignor , S. K. Allen , J. Boschung , A. Nauels , Y. Xia , V. Bex , & P. M. Midgley (Eds.), Climate Change 2013: The Physical Science Basis. Contribution of Working Group I to the Fifth Assessment Report of the Intergovernmental Panel on Climate Change. Cambridge University Press.

[mec16142-bib-0106] Thomaz, A. T. , & Knowles, L. L. (2020). Common barriers, but temporal dissonance: Genomic tests suggest ecological and paleo‐landscape sieves structure a coastal riverine fish community. Molecular Ecology, 29, 783–796. 10.1111/mec.15357 31958183

[mec16142-bib-0107] Tobler, M. , Kelley, J. L. , Plath, M. , & Riesch, R. (2018). Extreme environments and the origins of biodiversity: Adaptation and speciation in sulphide spring fishes. Molecular Ecology, 27, 843–859. 10.1111/mec.14497 29368386

[mec16142-bib-0108] Tossi, V. E. , Regalado, J. J. , Iannicelli, J. , Laino, L. E. , Burrieza, H. P. , Escandón, A. S. , & Pitta‐Álvarez, S. I. (2019). Beyond *Arabidopsis*: Differential UV‐B response mediated by UVR8 in diverse species. Frontiers in Plant Science, 10, 780. 10.3389/fpls.2019.00780 31275337 PMC6591365

[mec16142-bib-0109] Valdivia‐Carrillo, T. , García‐De León, F. J. , Blázquez, M. C. , Gutiérrez‐Flores, C. , & González‐Zamorano, P. (2017). Phylogeography and ecological niche modeling of the desert iguana (*Dipsosaurus dorsalis*, Baird & Girard 1852) in the Baja California Peninsula. Journal of Heredity, 108, 640–649. 28821185 10.1093/jhered/esx064

[mec16142-bib-0110] Vechio, F. D. , Prates, I. , Grazziotin, F. G. et al (2020). Rain forest shifts through time and riverine barriers shaped the diversification of South American terrestrial pit vipers (*Bothrops jararacussu* species group). Journal of Biogeography, 47, 516–526.

[mec16142-bib-0111] Velasco, J. A. , Martınez‐Meyer, E. , Flores‐Villela, O. et al (2016). Climatic niche attributes and diversification in Anolis lizards. Journal of Biogeography, 43, 134–144.

[mec16142-bib-0112] Walker, S. I. , & Davies, P. C. W. (2012). The algorithmic origins of life. Journal of the Royal Society Interface, 10, 20120869. 23235265 10.1098/rsif.2012.0869 PMC3565706

[mec16142-bib-0113] Wan, T. , Oaks, J. R. , Jiang, X.‐L. , Huang, H. , & Knowles, L. L. (2021). Differences in Quaternary co‐divergence reveals community‐wide diversification in the mountains of southwest China varied among species. Proceedings of the Royal Society B: Biological Sciences, 288, 20202567. 10.1098/rspb.2020.2567 PMC7892402 33402075

[mec16142-bib-0114] Weir, J. T. , Faccio, M. S. , Pulido‐Santacruz, P. , Barrera‐Guzmán, A. O. , & Aleixo, A. (2015). Hybridization in headwater regions, and the role of rivers as drivers of speciation in Amazonian birds. Evolution, 69, 1823–1834. 10.1111/evo.12696 26095719

[mec16142-bib-0115] Whipple, K. X. , & Tucker, G. E. (1999). Dynamics of the stream‐power river incision model: Implications for height limits of mountain ranges, landscape response timescales, and research needs. Journal of Geophysical Research: Atmospheres, 104, 17661–17674. 10.1029/1999JB900120

[mec16142-bib-0116] Winkler, N. S. , Matus, A. P. , Villena, Á. A. , & Thiel, M. (2017). Seasonal variation in epifaunal communities associated with giant kelp (*Macrocystis pyrifera*) at an upwelling‐dominated site. Austral Ecology, 42, 132–144.

[mec16142-bib-0117] Wood, T. E. , Takebayashi, N. , Barker, M. S. , Mayrose, I. , Greenspoon, P. B. , & Rieseberg, L. H. (2009). The frequency of polyploid speciation in vascular plants. Proceedings of the National Academy of Sciences of USA, 106, 13875–13879. 10.1073/pnas.0811575106 PMC2728988 19667210

[mec16142-bib-0118] Zeng, X. , Yuan, H. , Dong, X. , Peng, M. , Jing, X. , Xu, Q. , Tang, T. , Wang, Y. , Zha, S. , Gao, M. , Li, C. , Shu, C. , Wei, Z. , Qimei, W. , Basang, Y. , Dunzhu, J. , Li, Z. , Bai, L. , Shi, J. , … Nyima, T. (2020). Genome‐wide dissection of co‐selected UV‐B responsive pathways in the UV‐B adaptation of qingke. Molecular Plant, 13, 112–127. 10.1016/j.molp.2019.10.009 31669581

